# Retinal ganglion cell loss in kinesin-1 cargo Alcadein α deficient mice

**DOI:** 10.1038/s41419-020-2363-x

**Published:** 2020-03-03

**Authors:** Yuki Nakano, Kazuyuki Hirooka, Yoichi Chiba, Masaki Ueno, Daiki Ojima, Md Razib Hossain, Hiroo Takahashi, Tohru Yamamoto, Yoshiaki Kiuchi

**Affiliations:** 1 Department of Ophthalmology, Kagawa University Faculty of Medicine, Kagawa, Japan; 2 Department of Ophthalmology and Visual Science, Graduate School of Biomedical Sciences, Hiroshima University, Hiroshima, Japan; 3 Department of Inflammation Pathology, Kagawa University Faculty of Medicine, Kagawa, Japan; 4 Department of Molecular Neurobiology, Kagawa University Faculty of Medicine, Kagawa, Japan

**Keywords:** Cell death in the nervous system, Neurodegeneration

## Abstract

Maintenance of retinal ganglion cells (RGCs) activity is relied on axonal transport conveying materials required for their survival such as neurotrophic factors. Kinesin-1 undergoes anterograde transport in axons, and Alcadein α (Alcα; also called calsyntenin-1) is a major cargo adaptor protein that can drive kinesin-1 to transport vesicles containing Alcα. The long-term effects of Alcα-deficiency on retinal morphology and survival of RGCs during postnatal development were examined in Alcα knockout mice. At 1.5, 3, 6, and 15 months postnatal, the number of retrogradely labeled RGCs was determined in flat-mounted retinas of Alcα-deficient and wild-type mice. Retinal damage was assessed histologically by determining the retinal thickness. Intraocular pressure (IOP) was measured with a Tonolab tonometer. At 1.5 months postnatal, the number of retrogradely labeled RGCs was not different between wild-type and Alcα-deficient mice. However, at 3, 6, and 15 months postnatal, the number of RGCs was significantly lower in Alcα deficient mice than those of wild-type mice (143 ± 41.1 cells/mm^2^ vs. 208 ± 28.4 cells/mm^2^, respectively, at 3 months; *P* < 0.01). No differences were seen in retinal thickness or IOP between the two types of mice at any postnatal age. Alcα-deficient mice showed spontaneous loss of RGCs but no elevation in IOP. These mice mimic normal-tension glaucoma and will be useful for investigating the mechanism of neurodegeneration in this disorder and for developing treatments for RGC loss that does not involve changes in IOP.

## Introduction

Glaucoma involves axon pathology of retinal ganglion cells (RGCs) and is more commonly seen in older people and those with elevated intraocular pressure (IOP)^1^. However, some patients exhibit disease progression even when their IOP is normal or controlled with medication. The mechanism of neurodegeneration in glaucoma is not clear. Multiple stimuli induce neuronal apoptosis, which may be reversed by neurotrophic factors that promote neuronal development and survival^2^. RGC survival is mediated by brain-derived neurotrophic factor (BDNF), nerve growth factor (NGF), ciliary neurotrophic factor (CNTF), and glial cell line-derived neurotrophic factor (GDNF)^3^. Intact axonal transport is prerequisite for transducing such trophic signals, and impaired axonal transport is recognized as an initial pathological event commonly observed in neurodegenerative disorders^4^. In various glaucoma models, deficits in axonal transport were observed after elevation of IOP^5^, however, it is still unclear whether attenuation of axonal transport could be a cause for RGC degeneration.

Kinesin-1 is a major molecular motor employed for fast anterograde axonal transport. Kinesin-1 is composed of two heavy chains (KIF5) and two light chains (KLC); KIF5 contains motor activity, and KLCs associate with KIF5 motors. Kinesin-1 is kept inactive in a cell by maintaining auto-inhibited state, which can be released by simultaneous interaction of cargo molecules with KIF5 tail domain and KLC^6^. Alcadein α (Alcα; also called calsyntenin-1) is a member of neuronal type I single-pass transmembrane protein Alcadeins (also called calsyntenins), whose primary structures are evolutionarily conserved^7,8^. Alcα directly binds to kinesin light chain (KLC) through its C-terminal cytoplasmic region and is transported by kinesin-1^9^. Furthermore, Alcα indeed activates kinesin-1 by itself; Alcα can release the auto-inhibited state of kinesin-1 by itself only through the ~10 amino acids length W-acidic motif structure in the C-terminal cytoplasmic region of Alcα, forcing kinesin-1 to be activated for anterogradely transporting Alcα-containing vesicles^10,11^. It means that Alcα-containing vesicles are transported by kinesin-1 in priority to another vesicles, suggesting that Alcα would play an important role in transporting molecules that have to be preferentially delivered. These characteristic features of Alcα in axonal transport prompted us that Alcα deficiency could adversely affect homeostasis of neurons. In fact, in vitro studies showed that attenuation of Alcα function by introducing dominant-negative Alcα or siRNA into cultured cells reduces axonal transport of amyloid precursor protein (APP) to increase cytotoxic Aβ generation^9,12^, and recent in vivo study showed that Alcα-deficient mice eventually exhibited augmentation of amyloidogenic processing of endogeneous APP sufficient to lead pathologic feature of Alzheimer’s disease such as amyloid plaque formation^13^. Given that APP is not the sole protein of which transport is affected by Alcα, these observations collectively suggest that Alcα-deficiency might induce vulnerability of neurons by attenuating efficient traffic of necessary factors, which could finally lead to their degeneration in particular neurons.

Here we found and report progressive loss of RGCs in Alcα-deficient mice. We examined the long-term effect of Alcα-deficiency on retinal morphology and discuss on the role of Alcα in survival of RGCs during postnatal development.

## Materials and methods

### Experimental animals

All animal investigations were performed according to the guidelines for animal experimentation of the Kagawa University Faculty of Medicine and adhered to the ARVO Statement for the Use of Animals in Ophthalmic and Vision Research. The mice were kept in a standard animal room with a 12-h light–dark cycle and free access to food and water. Generation of Alcα knockout mice was described elsewhere^13^. Briefly, mouse genomic DNA including the first exon of *CLSTN1* (the gene encoding Alcα) was obtained from a C57BL/6 BAC clone (Invitrogen) and used to prepare a targeting vector. The vector was constructed by replacing the coding sequence with a *LacZ-pA-PGK-Neo-pA* cassette from the DT-A/LacZ/Neo plasmid; the vector was then electroporated into TT2 embryonic stem cells^14^. PCR with the following primers was performed to identify successful recombinants: 5′-ACCGCTTCCTCGTGCTTTACGGTATC-3′ and 5′-TAAGAACCTATTTAACAGGGGCTAGC-3′. Knockout mice were backcrossed to the C57BL/6 background for more than 10 generations. The PGK-Neo region of the cassette was removed by crossing these mice to transgenic C57BL/6 mice ubiquitously expressing flippase^15^. The wild-type allele and floxed *LacZ-pA-pA* allele were identified with PCR using the following primers: 5′-CGGGGTCTGGGCCGCGCGAGGTAA-3′ for wild type (416 bp); 5′-CGGGGTCTGGGCCGCGCGAGGTAA-3′ and 5′-GCTGGCTGCCATGAACAAAGGTTGG-3′ for *LacZ-pA-pA* (1224 bp).

### Histology

The mice of indicated age and genotypes were sacrificed following an intraperitoneal overdose of pentobarbital, and eyes were enucleated immediately and fixed in 4% paraformaldehyde at room temperature. Next, the anterior segments, including the lens, were removed. The posterior eyecups were embedded in paraffin, and 5-μm-thick sections, which contained the full length of the eyecup from the superior to inferior surface along the vertical meridian through the optic nerve head, were cut using a microtome. Each eye was mounted on a glass slide coated with silane and stained with hematoxylin and eosin. The thickness of the sclera was measured to confirm that the sections were not oblique.

Images were taken of five randomly selected sections per eye, within 0.5 mm of the optic disc. One investigator blinded to the genotype of the mice performed all light microscopic assessments (magnification; 10 × 100; Olympus BX-51, Olympus Inc., Tokyo, Japan) and determined the thickness of the inner plexiform layer (IPL), inner nuclear layer (INL), outer plexiform layer (OPL), and outer nuclear layer (ONL).

In situ hybridization of Alcα was performed essentially as described^16^. Briefly, fixed and cryoprotected eyes were embedded in OCT compound (Sakura Finetech, Tokyo, Japan) and sectioned into serial 20-µm coronal sections on a CM3000 cryostat (Leica Microsystems, Wetzler, Germany). The resultant sections were post-fixed, washed three times with PBS, and incubated in 1 µg/ml Proteinase K (Roche Applied Science) in 6.25 mM EDTA pH 8.0 (Dojindo Laboratories, Kumamoto, Japan) and 50 mM Tris pH 7.5 (Wako Pure Chemical Industries, Osaka, Japan) at RT for 5 min. The sections were re-fixed, washed with PBS, and acetylated in 1.33% triethanol amine (Sigma-Aldrich; St. Louis, MO), 0.75% acetic anhydride solution (Wako Pure Chemical Industries) at RT for 10 min. The acetylated sections were washed with PBS and incubated in hybridization buffer (50% formamide (Sigma-Aldrich), 0.25 mg/ml Yeast RNA (Sigma-Aldrich), 0.5 mg/ml herring sperm DNA (Roche Applied Science), 5x Denhard’s (Sigma-Aldrich), 5x SSC (0.75 M NaCl, 75 mM sodium citrate, pH 7.0)) at RT for 2 h, then with digoxigenin-labeled mouse Alcα cRNA probe in hybridization buffer at 72 °C for 16 h. The hybridized sections were washed in 5x SSC at 72 °C for 10 min and then in 0.2x SSC for 1 h. The washed sections were incubated with 10% heat-inactivated goat serum (Roche Applied Science) in 100 mM Tris pH 7.5, 0.15 M NaCl solution at RT for 1 h, followed by incubation with alkaline phosphatase-conjugated anti-digoxigenin antibody (Roche Applied Science) in the same solution at 4 °C overnight. The sections were washed with 100 mM Tris pH 7.5, 0.15 M NaCl solution, then with 100 mM Tris pH 9.5, 0.1 M NaCl, 50 mM MgCl_2_ solution, followed by incubation with NBT/BCIP (Roche Applied Science) in the same solution containing 0.24 mg/mL levamisole (Sigma-Aldrich) at RT in the dark. The reaction was stopped by immersing the sections in PBS-5 mM EDTA.

To detect Alcα in RGCs by immunohistochemistry, anti-Alcα antibody^9,17^ was used and visualized by donkey secondary antibodies (Jackson Immuno Research Laboratories, West Grove, PA) as described^16^. After washing the sections with PBS, the slides were mounted with Shandon Immu-Mount (Cat #9990402; Thermo Fisher Scientific, Waltham, MA) and observed by fluorescence microscopy with ×20 objective and ×10 eyepiece lens followed by merging respective images (BZ-9000; Keyence, Osaka, Japan).

### Western blot analysis

To detect Alcα in RGCs by western blotting, isolated retinal tissues were homogenized in RIPA buffer containing 1% SDS and protease inhibitor cocktail (5 µg/ml chymostatin, 5 µg/ml leupeptin, and 5 µg/ml pepstatin), and centrifuged (15,000 rpm for 15 min at 4 °C). The supernatants were used for western blot analysis as described^16^. Briefly, equal amounts of total protein (1 μg) were loaded for each sample and run on a 12% polyacrylamide gel. The proteins were then transferred onto a PVDF membrane (Immobilon-P membrane, Millipore, Burlington, MA) using a wet tank system (BIO CRAFT, Tokyo, Japan). The membranes were blocked for 30 min at RT in 5% skim milk in TTBS (20 mM Tirs-HCl pH 7.5, 150 mM NaCl, 0.005% Tween-20, 5 mM CaCl_2_). After washing with TTBS, the membranes were incubated with the primary antibodies diluted in TTBS overnight at 4 °C. After washing with TTBS, the membranes were treated with horseradish peroxidase (HRP)-conjugated secondary antibodies in TTBS for 1 h at RT. The following antibodies were used; anti-Alcα (guinea pig, 1:5000)^9^, anti-α tubulin (mouse, 1:5000, Abcam, Cambridge UK), HRP-conjugated anti-guinea pig IgG (donkey, 1:15000, Jackson Immuno Research Laboratories), and HRP-conjugated anti-mouse IgG (sheep, 1:15000, GE Healthcare, Chicago, IL). Immunoreactive bands were detected by ECL Western Blotting Detection System (GE Healthcare), and captured with luminescent image analyzer (ImageQuant LAS 4010, GE Healthcare).

### Retrograde labeling of RGCs

Mice were anesthetized with intraperitoneal injection of 0.75 mg/kg medetomidine, 4.0 mg/kg midazolam, and 5.0 mg/kg butorphanol before surgical procedures. The skull was exposed and kept clean and dry. Bregma was marked, and then a small window (2.0 mm deep from the surface of the skull; 2.92 mm behind the bregma on the anteroposterior axis; 0.5 mm lateral to the midline) was drilled into the skull in both hemispheres. Using a Hamilton syringe (Hamilton Co., Reno, NV), 1.0 μl of 3% Fast Blue (Polysciences Inc., Warrington, PA) was slowly injected into the superior colliculi on both sides. The skin over the wound was sutured, and antibiotic ointment was applied. Mice were sacrificed 7 days later.

### Tissue preparation and assessment of RGC survival

One week after Fast Blue injection, mice were sacrificed following an overdose of pentobarbital. RGC density was assessed in whole, flat-mounted retinas. Eyes were enucleated and fixed in 4% paraformaldehyde for 10 h at room temperature. The posterior eyecups were left in place after the anterior segments were removed. Next, four radial cuts were made in the periphery of each eyecup, and the retina was carefully removed from the retinal pigment epithelium. The retina was removed from other underlying structures, flattened by making four radial cuts, and mounted on a gelatin-coated glass slide. Labeled RGCs were visualized with fluorescence microscopy (Olympus BX-51/DP-72, Olympus, Tokyo, Japan) and an ultraviolet filter (excitation filter, 330–385 nm; barrier filter, 420 nm). RGCs were counted in 12 microscopic fields of the retina in two regions per quadrant at two different eccentricities from the optic disc: 0.3–0.8 mm (central) and 1.2–1.7 mm (peripheral). Image J software (Version 1.51a, Wayne Rasband National Institutes of Health, USA) was used to determine the total number of RGCs per eye.

### Immunohistochemistry for Brn-3a

Eyes were enucleated at 3, 8, and 15 months after birth, fixed in 4% paraformaldehyde, and embedded in paraffin. Retinal sections (5 μm) were rinsed twice in 100% ethanol for 5 min each, and then rinsed for 3 min each with 95% ethanol and then 90% ethanol. The sections were rinsed in phosphate-buffered saline (PBS, pH 7.4) three times for 10 min each and then incubated in 0.3% Triton X-100 in PBS for 1 h. After further washing with PBS (three times for 10 min each), sections were blocked in 3% normal horse serum and 1% bovine serum albumin (BSA) in PBS for 1 h. Sections were incubated overnight at 4 °C in the primary antibody, rabbit polyclonal antibody against human Brn-3a (3:100; Catalogue Number: AB5945, Merck KGaA, Darmstadt, Germany) in 3% BSA in PBS. Sections were washed three times for 5 min each in PBS and then incubated at room temperature for 30 min in Histofine Simple Stain MAX PO R (Nichirei Bioscience Inc., Tokyo, Japan) as the secondary antibody. Sections were washed three times for 5 min each in PBS and then incubated for 5 min at room temperature in Histofine DAB (Nichirei Bioscience Inc.) to allow color development. Sections were washed for 5 min in water, counterstained for 1 min at room temperature in Mayer’s Hematoxylin Solution, and washed for 5 min in water. Images of stained sections were acquired using ×40 objective lenses (DXM 1200; Nikon, Tokyo, Japan). Adobe PhotoShop v. 5.0 was used to adjust the brightness and contrast of the images.

### IOP measurement

Mice were anesthetized by intraperitoneal injection of 0.75 mg/kg medetomidine, 4.0 mg/kg midazolam, and 5.0 mg/kg butorphanol, and then IOP was measured with a TonoLab tonometer (TioLat, Inc., Helsinki, Finland). We calculated the mean of six readings using the optimal variability grade.

### Statistical analysis

All data are shown as the mean ± standard error of the mean (SEM). All data were in a normal distribution, and variance was similar between the groups that are being statistically compared. Data were analyzed with an independent Student’s *t-*test or Student’s *t*-test, as appropriate. There were no exclusion criteria. Statistical analyses were performed with SPSS version 19.0 (SPSS Inc., Chicago, IL), and *P* < 0.05 was considered statistically significant. Randomization, blinding, and sample size estimation tests were not done for our animal studies.

## Results

### RGC loss in Alcα-deficient mice

We first examined the gross anatomy of 6-, 12-, and 24-month-old Alcα-deficient mice brains. Despite of above anticipation, including progressed AD pathogenesis of APP23 transgenic mice expressing human APP in Alcα-deficient background^13^ (APP23 was utilized for detecting amyloid plaque formation, since mouse Aβ does not make aggregation that is prerequisite for the plaque formation), we did not find apparent anatomical or pathological alteration between wild-type and Alcα-deficient mice brains in these age (data not shown). Then we switched our investigation to afferent neurons including RGCs.

**Fig. 1:**
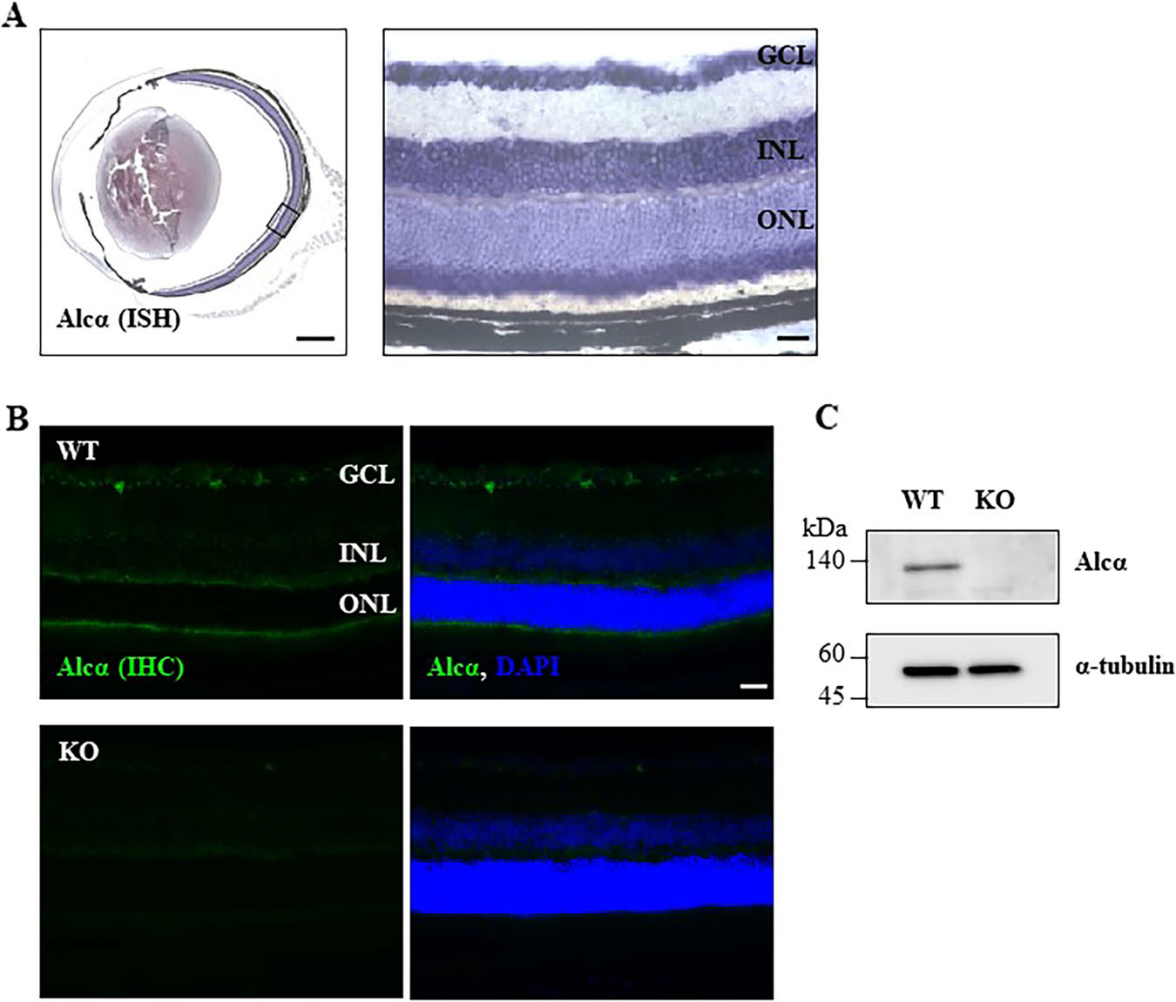
Expression of Alcα in the mouse retina. **a** Expression of Alcα mRNA in an eye of 5-month-old wild-type mice was detected by in situ hybridization (ISH). The magnified image in the boxed area of the left side image is shown on the right side. Alcα mRNA is readily detected in ganglion cells, cells in inner nuclear layer, and photoreceptors’ inner segment. GCL: ganglion cell layer, INL: inner nuclear layer, ONL: outer nuclear layer. Scale bar, 500 μm. **b** Expression of Alcα protein in an eye of 5-month-old wild-type (WT) and Alcα-deficient (KO) mouse was detected by immunohistochemistry (IHC). Alcα protein is readily detected in ganglion cells, photoreceptors’ inner segment, and outer peripheral layer. Alcα protein is not detected in the eye of Alcα-deficient mouse. Co-stained images with DAPI are shown on the right sides. GCL: ganglion cell layer, INL: inner nuclear layer, ONL: outer nuclear layer. Scale bar, 50 μm. **c** Expression of Alcα protein in a retina of 2-month-old wild-type (WT) and Alcα-deficient (KO) mouse was detected by western blotting. Alcα protein is not detected in the retinal lysate (1 μg) of Alcα-deficient (KO) mouse.

We verified expression of Alcα in retina of adult mice with in situ hybridization, immunohistochemistry, and western blotting. Alcα is expressed in retina including RGCs, which is not detected in Alcα knockout mice, confirming their Alcα-deficiency in the retina (Fig. 1).

**Fig. 2:**
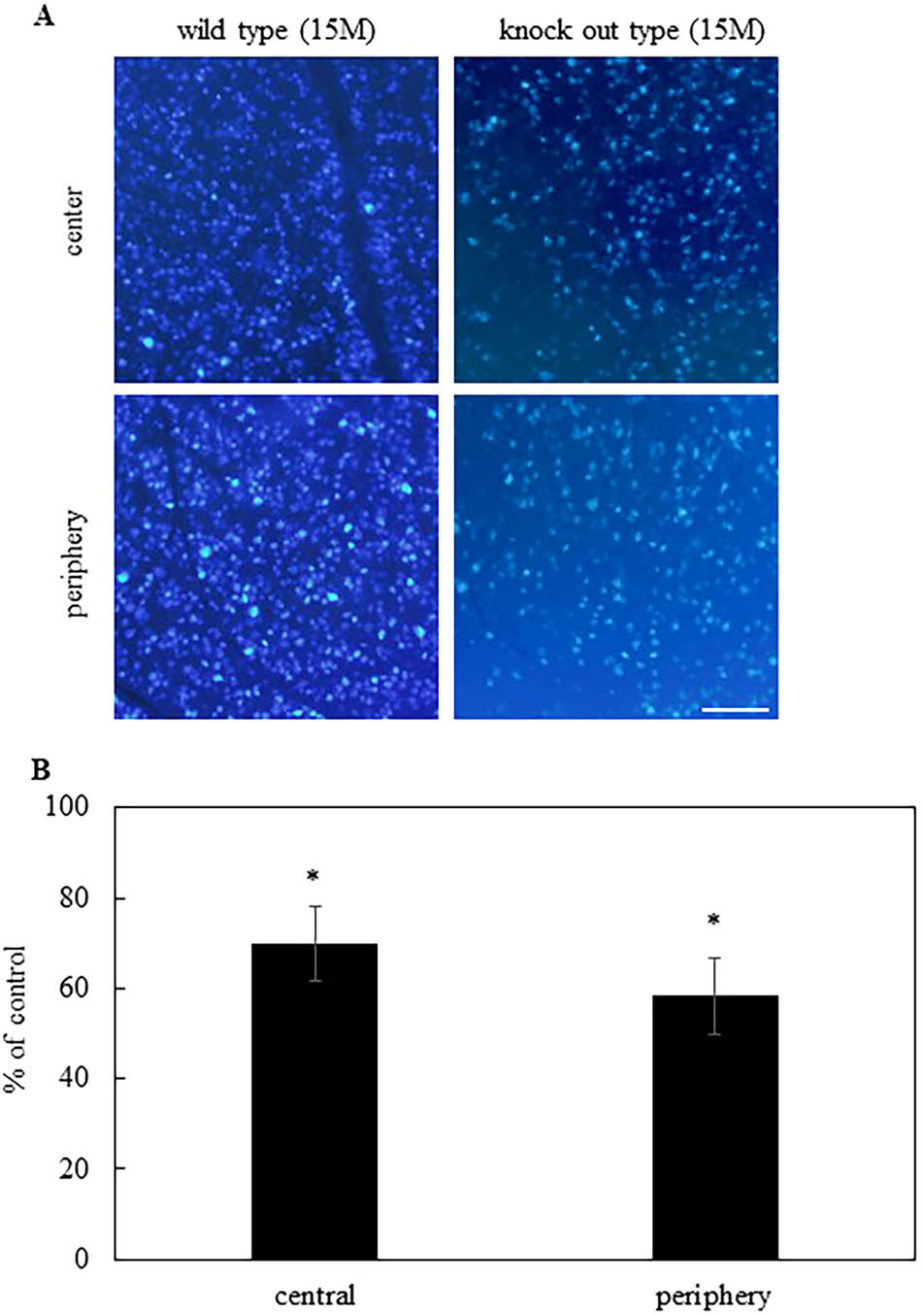
Retrograde labeling of RGCs in wild and knockout mice. **a** Typical micrographs of wild type at 15 months after birth and knockout mice at 15 months after birth. Micrographs of the central and peripheral areas were taken 0.3–0.8 mm and 1.2–1.7 mm from optic nerve head. **b** The number of RGCs were counted in the central (*n* = 8) and peripheral areas (*n* = 8). Result were expressed as the mean ± SEM. *P < 0.01 versus control (Student *t*-test). Scale bar, 100 μm.

We then examined if there is difference in RGCs between 15-month-old wild-type and Alcα-deficient mice by retrogradely labelling RGCs with Fast Blue bilaterally injected into their superior colliculi. Seven days after injection, numbers of labeled RGCs were counted (Fig. 2a). The numbers of RGCs in Alcα-deficient mice were shown as percentages compared with those of wild-type mice. Statistically significant difference was observed in their numbers of RGCs: numbers of labelled RGCs were significantly lower than those of wild-type mice both in the central (69.8 ± 8.3%, *P* < 0.01, *n* = 8) and peripheral retina (58.3 ± 8.3%, *P* < 0.01, *n* = 8) (Fig. 2b), indicating that Alcα-deficiency caused loss of RGCs at this age.

**Fig. 3:**
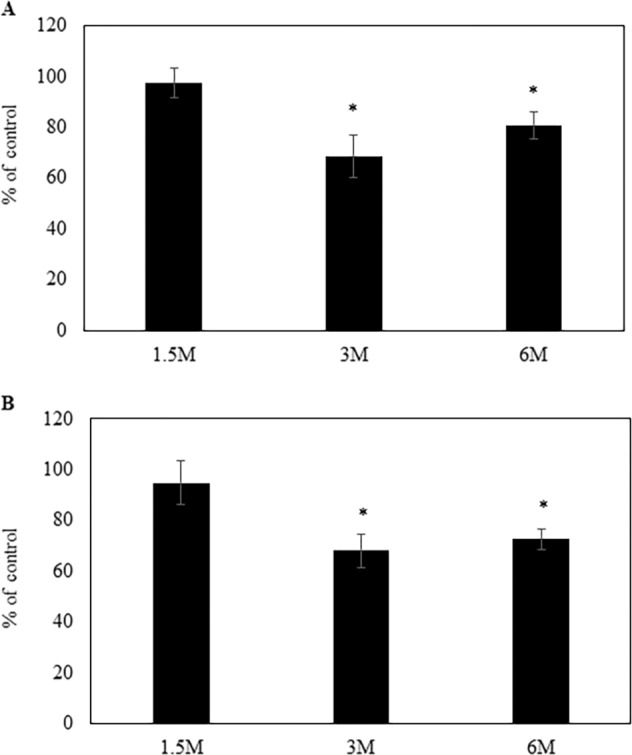
The number of RGCs of knockout mice at 1.5 (*n* = 8), 3 (*n* = 10), and 6 (*n* = 7) months after birth. **a** In the central retina, RGC was decreased 3 months after birth. **b** In the peripheral retina, reduction of number of RGCs was also detected at 3 months after birth. *P < 0.05 versus control (Student *t*-test).

We next verified the onset of the observed RGC loss. We examined the numbers of RGCs in Alcα-deficient mice at 1.5, 3, and 6 months old with the same retrograde labelling. RGC loss was not observed in 1.5-month-old Alcα-deficient mice, indicating that generation and maintenance of RGCs are unaltered until adolescent stage. However, significant RGC loss was observed at 3-month-old Alcα-deficient mice: numbers of labelled RGCs were significantly lower than those of wild-type mice both in the central (73.0 ± 8.4%, *P* = 0.01, *n* = 10) and peripheral retina (68.0 ± 6.5%, *P* < 0.01, *n* = 10) (Fig. 3). Statistically significant loss of RGCs was also observed at 6 months of age both in the central (79.3 ± 5.4%, *P* < 0.01, *n* = 7) and peripheral retina (73.0 ± 4.1%, *P* < 0.01, *n* = 7) (Fig. 3). These observations suggest that Alcα-deficiency-induced RGC loss is not due to developmental defect but due to deficit in maintaining RGC activity.

### Loss of Brn3a^+^ RGCs in Alcα-deficient mice

**Fig. 4:**
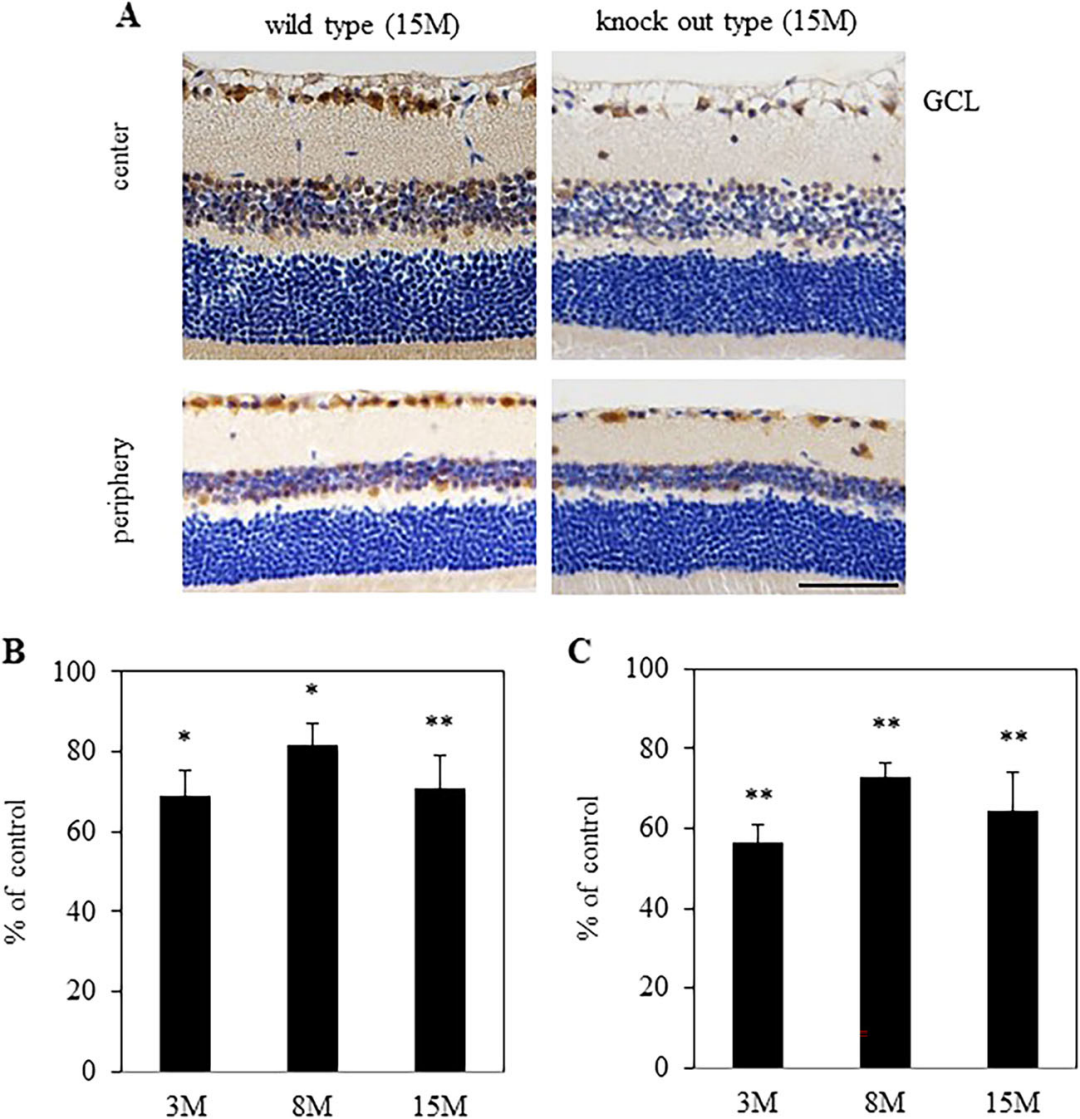
Evaluating the number of RGCs with Brn-3a immunostaining. **a** Typical micrographs of Brn-3a immunostaining in wild-type and knockout type at 15 months after birth. Micrographs of the central and peripheral areas were taken 0.3–0.5 mm and 1.4–1.6 mm from optic nerve head. The number of RGCs were counted with Brn-3a immunostaining in the central (**b**) and peripheral (**c**) areas. Result were expressed as the mean ± SEM. 3 M: *n* = 5, 8 M: *n* = 9, 15 M: *n* = 7. *P < 0.05, **P < 0.01 versus control (Student *t*-test). Scale bar, 50 μm.

Alcα possesses privileged activity that drives kinesin-1 by itself, and it is highly plausible that Alcα-deficiency should affect some aspects of axonal transport. Thus, in theory, it is hard to exclude the possibility that Alcα-deficiency might affect efficiency of retrograde labelling of RGCs with Fast Blue, even though fairly enough time (7 days) was allowed to label the cells, and no significant difference was observed in the numbers of labelled RGCs of 1.5-month-old mice. To verify the possibility, we directly counted the numbers of Brn3a-expressing RGCs in the retinal sections of wild-type and Alcα-deficient mice (Fig. 4a) at 3, 8, and 15 months of age (Fig. 4b, c). Brn3a is a transcription factor that is selectively expressed in great majority of RGCs in rodent retina^18,19^. Brn3a-positive RGCs were significantly decreased in Alcα-deficient mice at 3 months of age (central retina: 68.9 ± 6.5%, *P* = 0.01, *n* = 5; peripheral retina: 56.3 ± 4.7%, *P* < 0.01, *n* = 5), 8 months of age (central retina: 81.5 ± 5.6%, *P* = 0.01, peripheral retina: 72.9 ± 3.8%, *P* < 0.01, *n* = 9), and 15 months of age (central retina: 70.9 ± 8.1%, *P* < 0.01, peripheral retina: 64.7 ± 9.3%, *P* < 0.01, *n* = 7), compared with those of wild-type mice, confirming that Alcα-deficiency causes loss of RGCs.

### Thicknesses of other layers in retina were not significantly altered in Alcα-deficient mice

**Fig. 5:**
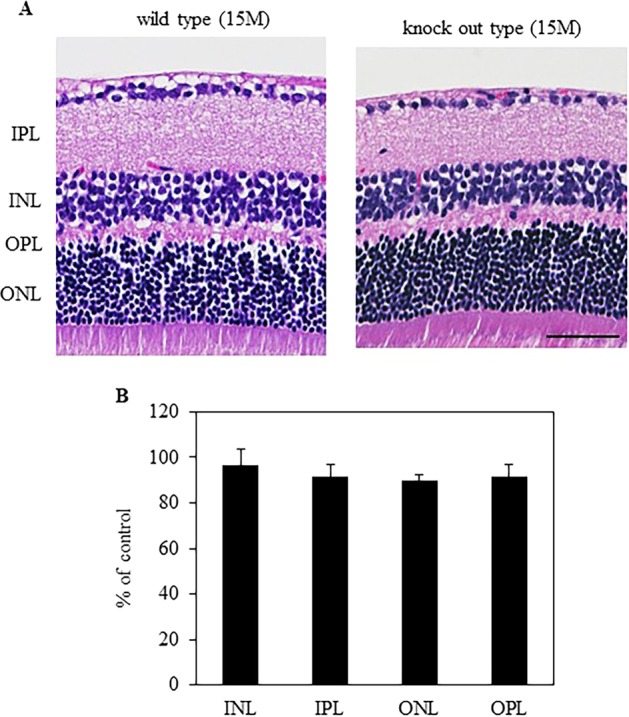
Retinal layer thickness analysis. **a** Typical micrographs with hematoxylin and eosin (HE) staining in wild type and knockout type at 15 months after birth. Mean thickness of inner plexiform layer (IPL), inner nuclear layer (INL), outer plexiform layer (OPL), and outer nuclear layer (ONL). **b** Result were expressed as the mean ± SEM. n = 6. Scale bar, 50μm.

Alcα is also expressed in other retinal cells (Fig. 1), of which deficiency might affect the maintenance of these cells. To verify the possibility, retinal layer thickness analysis was performed to check for degeneration of any of the other retinal neurons at the age of 15 months. The percent thickness in Alcα-deficient mice compared with control mice showed no statistically significant difference: 96.5 ± 7.2% (*P* = 0.63) for the IPL, 91.4 ± 5.6% (*P* = 0.21) for the INL, 89.8 ± 2.8% (*P* = 0.06) for the OPL, and 91.4 ± 5.6% (*P* = 0.23) for the outer nuclear layer (Fig. 5), suggesting that Alcα-deficiency selectively affects survival of RGCs in retina.

### IOP was not altered in Alcα-deficient mice

**Fig. 6:**
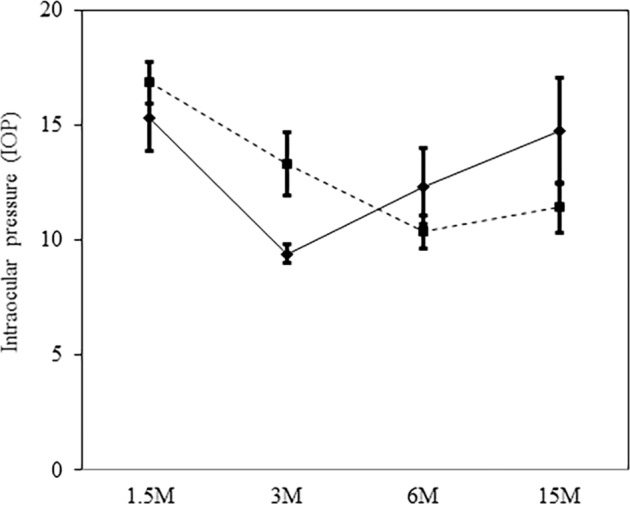
Intraocular pressure (IOP) in wild type and knockout type. Result were expressed as the mean ± SEM. There were no significantly differences in the IOP between wild-type and knockout mice. Wild type: broken line, knockout type: solid line. Wild type; 1.5 M: *n* = 10, 3 M: *n* = 10, 6 M: *n* = 20, 15 M: *n* = 8. Knockout type; 1.5 M: *n* = 8, 3 M: *n* = 10, 6 M: *n* = 6, 15 M: *n* = 6.

To verify the effect of Alcα on IOP, IOP of wild-type and Alcα-deficient mice was measured at various ages. We found no significant differences in IOP between wild-type and Alcα-deficient mice at any age (Fig. 6) (*P* = 0.07–0.37, *n* = 6–20), indicating that RGC loss in Alcα-deficient mice was independent of IOP.

## Discussion

Axonal transport plays a pivotal role in maintaining neuronal activity, and its deficit is one of common mechanisms underlying early-stage pathophysiology of various neurodegererative disorders. Alcα is an evolutionary conserved characteristic kinesin-1 cargo protein: that is not only transported by kinesin-1, but is also able to drive kinesin-1 to transport Alcα-containing vesicles. We examined Alcα-deficient mice in the context of neuroanatomy and degeneration, and found that RGCs were lost in these mice without elevation of IOP. Our studies added an evidence affirming importance of axonal transport on maintenance of RGCs, and further suggesting that Alcα plays a role necessary for RGC survival and may be closely associated with normal-tension glaucoma (NTG) pathology.

There are several possibilities how Alcα contributes RGC maintenance. Alcα is involved in anterograde axonal transport, and deficiency of Alcα could perturb homeostatic regulation of axonal transport including retrograde transport; since motors and adaptors required for retrograde transport have to be delivered to nerve terminals once. Target-derived neurotrophic factors are essential for survival of neurons including RGC, and it would be plausible that inefficient delivery of such trophic factors from the targets might be involved in loss of RGCs in Alcα-deficient mice. BDNF is reported as one of prominent target-derived trophic factor, of which administration protects RGCs from their death after nerve injury. We verified the amount of BDNF in the retina of Alcα-deficient mice, and found no statistically significant difference (data not shown). Ineffective delivery of other neurotrophic factors such as CNTF, GDNF, and NGF might contribute to the vulnerability of Alcα-deficient RGC. Another possible candidate is molecules preferentially transported with Alcα-containing vesicles. It was reported that axon guidance receptors Robo1 and Frizzled 3 are transported with Alcα in developing chick spinal commissural neurons^20^. Frizzled is a Wnt receptor, and canonical Wnt signaling is reportedly transduced into a population of adult RGCs^21^. Alcα-deficiency might cause inefficient transport of such molecules to reduce tolerance of RGCs to external stress leading loss of RGCs. Further investigation should be required for verifying these possibilities.

It has been shown that Alcα is involved in regulation of amyloidogenic processing of APP: attenuation of Alcα reduces transport of APP to augment cytotoxic Aβ production^9,12^, and Alcα-deficient mice exhibit enhanced amyloidogenic processing of APP to generate more Aβ sufficient for exacerbating AD related pathogenesis such as amyloid plaque formation^13^. Alzheimer’s disease is characterized by the death of hippocampal and cerebral cortical neurons due to oligomerized amyloid β (Aβ) and tau protein in the brain^22,23^. Several studies have suggested a relationship between glaucoma and neurodegenerative diseases such as Alzheimer’s disease^24,25^. Animal models have been used to test the relationship between Aβ and glaucoma. Wang et al.^26^ reported that Aβ is toxic to RGCs in vivo and showed that Aβ is present adjacent to apoptotic RGCs, suggesting a possible role for Aβ neurotoxicity in the development of RGC death in glaucoma. Kipfer-Kauer et al.^27^ reported caspase activation and abnormal APP processing in RGCs of rats with induced chronic ocular hypertension. It was recently reported that Aβ peptides primarily inhibit glutamate re-uptake to sensitize neurons for stimulations getting them more hyperactive^28^. RGC is also susceptible to excitotoxicity^29^, and such Aβ-mediated hyperactivity might be a cause of RGC loss in Alcα-deficient mice. Further investigation aimed for understanding the mechanisms of regulation of Aβ generation in the eye may become important step for understanding NTG pathogenesis.

Glaucoma is an age-related neurodegenerative disorder. In Alcα-deficient mice, generation and maintenance of RGCs appeared normal until adolescent stage, suggesting that Alcα-deficient mice exhibited no apparent effect on development and maturation, partly reflecting human glaucoma. Loss of RGCs was observed from 3 months of age, and the apparent ratio of lost RGCs looks rather constant (~30%) along with age, although numbers of eyes exhibiting lower than mean minus 2 standard deviation. of wild-type mice seem to increase in older mice. It is obscure why Alcα-deficient mice showed such step-wise loss of their RGC. One of possibilities would be that some populations of RGCs are more susceptible to circumstantial stress accumulated and/or become evident by the age. RGCs are not homogeneous but consist of ~30 subtypes, of which function and dendritic structures are different^30^. Some of these subtypes of RGCs may be more vulnerable than other subtypes in Alcα-deficient condition. Given that Wnt signaling is transduced into a subpopulation of adult RGCs^21^, some of molecules involved in such trophic mechanism may be inefficiently transported without Alcα, and the most labile subpopulations of RGCs to those deficiencies might become degenerated around those time points. It should not be also excluded that 15 months old may not be old enough, and more RGC loss might occur in much older mice. Further investigation including analyzing older mice would be required to verify the possibilities.

In conclusion, Alcα-deficient adult mice showed RGC loss that was independent of elevated IOP. We believe that this animal model could be utilized as a tool for investigating the mechanisms of neurodegeneration in NTG and for developing treatments for IOP-independent RGC loss.
